# Miniaturization and Automation Protocol of a Urinary Organic Acid Liquid-Liquid Extraction Method on GC-MS

**DOI:** 10.3390/molecules28155927

**Published:** 2023-08-07

**Authors:** Masauso Moses Phiri, Elmarie Davoren, Barend Christiaan Vorster

**Affiliations:** 1 Department of Pathology and Microbiology, School of Medicine, University of Zambia, Lusaka 10101, Zambia; 2 Centre for Human Metabolomics, North-West University, Potchefstroom 2531, South Africa

**Keywords:** organic acids, liquid-liquid extraction, automation, miniaturization, method validation

## Abstract

The aim of this study was to improve the extraction method for urinary organic acids by miniaturizing and automating the process. Currently, manual extraction methods are commonly used, which can be time-consuming and lead to variations in test results. To address these issues, we reassessed and miniaturized the in-house extraction method, reducing the number of steps and the sample-to-solvent volumes required. The evaluated miniaturized method was translated into an automated extraction procedure on a MicroLab (ML) Star (Hamilton Technologies) liquid handler. This was then validated using samples obtained from the ERNDIM External Quality Assurance program. The organic acid extraction method was successfully miniaturized and automated using the Autosampler robot. The linear range for most of the thirteen standard analytes fell between 0 to 300 mg/L in spiked synthetic urine, with low (50 mg/L), medium (100 mg/L), and high (500 mg/L) levels. The correlation coefficient (r) for most analytes was >0.99, indicating a strong relationship between the measured values. Furthermore, the automated extraction method demonstrated acceptable precision, as most organic acids had coefficients of variation (CVs) below 20%. In conclusion, the automated extraction method provided comparable or even superior results compared to the current in-house method. It has the potential to reduce solvent volumes used during extraction, increase sample throughput, and minimize variability and random errors in routine diagnostic settings.

## 1. Introduction

Organic acids are low molecular weight, water-soluble compounds that play a vital role in human metabolic processes [1,2]. Urine contains a wide variety of organic acids, originating from various sources such as normal and abnormal metabolism, drug metabolism, and xenobiotics [3]. These organic acids serve as intermediate products in numerous metabolic pathways, including those involving amino acids, sugars, biogenic amines, steroids, lipids, and more [4]. Additionally, they are associated with a subgroup of inborn errors of metabolism (IEM), which are characterized by the accumulation of organic acids, primarily in urine and other bodily fluids [1]. Consequently, conducting comprehensive and quantitative analysis of these metabolites, through targeted and untargeted approaches, can provide valuable insights into the pathophysiological and physiological conditions of various metabolic pathways, as well as their interrelationships [5].

The major challenge in analyzing urinary organic acids using gas chromatography mass spectrometry (GC-MS) is the need for proper sample preparation and derivatization before analysis. Sample preparation plays a crucial role in ensuring adequate coverage of metabolites, producing high-quality results, and enabling accurate biological interpretation of the data [6]. In 1980, Tanaka et al. [7] described a sample preparation method for determining organic acids in urine samples using GC and GC-MS. The method involved several key steps: acidifying the sample, performing successive liquid-liquid extraction (LLE) using ethyl acetate, vigorously shaking the mixture, combining the organic layers in a separate tube, drying the extract, and finally, derivatizing it. Since then, the fundamental principles of this method have remained largely unchanged, with only minor modifications [2,3].

Nevertheless, there are variations among laboratories in terms of solvent and internal standard selections, solvent ratios, sample-to-solvent ratios, mixing times, and other factors. This lack of a conventional and standardized method contributes significantly to the inconsistencies observed between different laboratories [2,8]. There have been significant advancements in robotic hardware and computer software, coupled with a reduction in their prices, which have greatly facilitated the automation of the sample preparation process [9]. Automation not only minimizes and enhances the consistency of repetitive tasks previously carried out by analysts but also reduces pre-analytical errors in the laboratory, improves clinical workflow, and decreases turnaround time and cost [9].

Despite the potential benefits, little progress has been made in automating the liquid-liquid extraction (LLE) step for urinary organic acids [10]. This lack of progress can be attributed to several challenges within the extraction protocol itself, such as the acidification of the sample, ensuring efficient recovery of the extracted organic solvent, rotary mixing, and centrifugation steps, removing residual water from the organic solvent, evaporating the solvent during the concentration step, and performing derivatization [9].

Nonetheless, overcoming these challenges would pave the way for a fully miniaturized and automated extraction protocol for urinary organic acids. Such a protocol would offer advantages such as high sample throughput, reduced labor costs, decreased random errors, improved precision, and long-term cost savings [11,12,13,14]. Therefore, the objective of this study was to develop an automated method for extracting organic acids by reassessing and optimizing the existing manual extraction method and automating it.

## 2. Results and Discussion

### 2.1. Miniaturization of In-House Sample Preparation Method

The study aimed to reassess and optimize the in-house organic acid extraction method used at the laboratory for inborn errors of metabolism for automation. The objective was to automate the extraction protocol with specific requirements, including using a sample volume of 500 μL or less, performing the extraction in a 2.0 mL glass vial, and using two solvents that are good extractants of organic acids. Additionally, the sample-to-solvent ratio was not to exceed a 1:4 ratio to maintain a total volume of less than 1.8 mL in the vial. There was a need to eliminate centrifugation while ensuring good extraction efficiency through multiple cumulative extractions. The goal was to fully automate the sample clean-up step before solvent evaporation in the sample concentration process.

The experimental workflow involved selecting an initial suitable miscible solvent to rapidly extract organic acids, especially very polar ones. Then, a suitable immiscible solvent was chosen to ensure effective isolation of organic acids, quick evaporation, and clear phase separation, eliminating the need for centrifugation. The solvent ratios and volumes were optimized and miniaturized to small volumes. The miniaturized organic acid protocol was then transformed into a fully automated extraction procedure using a liquid AutoSampler. 

The miniaturized manual extraction method was developed involving a two-phase extraction system using two optimal solvents, acetonitrile and ethyl acetate, which were found to be efficient in the extraction of urinary organic acids, enabling efficient and rapid extraction. Based on extraction efficiencies, acetonitrile was selected as the optimal initial one-phase extractant. Acetonitrile being a polar solvent, miscible with water and having higher dielectric constants compared to immiscible, was observed to extract the more polar compounds better. Therefore, after solvent volume optimizations, 300 μL was used in the initial extraction step in the miniaturized method. Ethyl acetate was selected as the solvent of choice from the immiscible organic solvents. Ethyl acetate demonstrated superior extraction efficiencies and extraction repeatability for organic acids compared to other investigated immiscible solvents. It offered various advantages, such as being ready to use, having good viscosity, leading to clear phase separation with urine, having a relatively low solubility index with water for rapid solvent evaporation, and possessing lower density than water for upper phase extraction. Additionally, it was able to extract acetonitrile from the aqueous phase when more than three times (1200 μL) its volume was added to the sample-acetonitrile mixture. These two solvents fulfilled the requirements for the initial one-phase and secondary two-phase extraction solvents.

The miniaturized method was then compared to the in-house method by assessing the coefficient of variation (CV) and the extraction efficiency of the spiked organic acids. Table 1 presents a summary of the calculated mean recovery and CV values for thirteen organic acids and sorbitol.

Organic acids with recoveries above 100% indicate that they were extracted in higher quantities relative to the internal standard. Several organic acids demonstrated good recoveries and were comparable to the in-house method. Phenylacetic acid, ethylmalonic acid, methylmalonic acid, and stearic acid exhibited recoveries above 100% for the miniaturized method, which were higher than those obtained by the in-house method. 4-Phenylbutyric acid and fumaric acid showed similar recoveries between both methods. On the other hand, adipic acid, succinic acid, and glutaric acid displayed lower recoveries for the miniaturized method compared to the in-house method. Citric acid, glycolic acid, lactic acid, and glutaconic acid had recoveries of less than 50% for the miniaturized method but performed better with the in-house method.

Acceptable coefficient of variation (CV) values were considered to be below 20%, while values above 20% were deemed unacceptable. From Table 1, it can be observed that citric acid and glutaconic acid exhibited the highest degree of variability for the miniaturized method. However, glutaconic acid still had a lower CV value compared to the in-house method. The in-house method had higher CVs for glycolic acid and lactic acid when compared to the miniaturized method. Notably, the miniaturized method demonstrated better repeatability than the in-house method, as most organic acids had CVs below 20%.

According to Blau et al. (2014), the extraction efficiencies of highly polar organic acids remain challenging as they tend to prefer the aqueous phase. This explains why citric acid exhibited the lowest recovery and repeatability for both methods. Interestingly, the extraction efficiencies of less polar organic acids were unaffected by the volume of solvent used. This indicates that the extraction efficiencies of organic acids are not necessarily dependent on the amount of solvent employed but are more influenced by their own polarity.

### 2.2. Method Validation

#### 2.2.1. Linearity

Table 2 presents the linear range and correlation coefficient (r) observed for each analyte extracted using the miniaturized method, ranging from 0 to 500 mg/L. The linear ranges for most of the organic acids fell within 0 to 300 mg/L. Most analytes exhibited a correlation coefficient (r) higher than 0.995, except for succinylacetone, malonic acid, and glycolic acid, which had a correlation coefficient below the acceptable value of 0.995. Thus, the method demonstrated its capability to analyze most organic acids within these concentrations without requiring dilution. Further optimizations is needed for the three organic acids that fell below the acceptable values according to the guidelines of the International Council on Harmonization (ICH).

#### 2.2.2. Precision Study

The replication experiments were conducted to assess the precision of the automated organic acid method. Table 3 provides an overview of the coefficient of variation (CV) for all analytes at three concentration levels: 50 mg/L, 100 mg/L, and 500 mg/L. The repeatability data revealed a high level of agreement between successive measurements under the same conditions, as most organic acids exhibited CVs below 20%.

Table 3 indicates that certain organic acids achieved better CVs at higher concentrations (e.g., glutaric acid and glycolic acid), while others showed the opposite trend (e.g., ethylmalonic acid and fumaric acid). This variation could be attributed to differences in extraction efficiencies, the sensitivity of the GC-MS instrument, and/or peak integration.

Within-laboratory precision displayed greater variation compared to the repeatability results, likely due to variations in extractions across consecutive days of analysis. Only a few organic acids, specifically succinic acid, 4-phenylbutyric acid, ethylmalonic acid, stearic acid, fumaric acid, methylmalonic acid, and adipic acid, achieved within-lab precision below 20%. However, it is important to note that the precision of the extraction process was considered more critical since it can be compensated for using external calibrators or response factors.

#### 2.2.3. Accuracy Study

EQA samples were utilized to compare the automated method with the in-house method, focusing only on organic acids included in the EQA scheme. The results of the regression statistics and the number of systematic errors in the methods’ outcomes are presented in Table 4. The test method was plotted on the *y*-axis, while the comparative method was plotted on the *x*-axis. The correlation coefficient (r) for all analytes in the automated method exceeded 0.99, except for 4-hydroxybutyric acid (0.96), 3-hydroxyglutaric acid (0.378), 2-hydroxyglutaric acid (0.98), methylcitric acid (0.98), N-hexanoylglycine (0.97), mevalonic acid (0.97), and pyroglutamic acid (0.98). The obtained r values for the automated method indicated a strong fit between the observed results and the expected EQA results, demonstrating the method’s accuracy.

The r values obtained from the automated method indicated a lower level of dispersion compared to the in-house method when analyzing the EQA results. Since r depends on the distribution and range of the data, the method demonstrated a good level of agreement with the expected EQA results. The reliable determination of the slope (proportional error) and intercept (constant error) using the automated method provided confidence in assessing other errors within the data. The constant error, estimated from the y-intercept of the regression statistics, ranged from −5.75 to 5.66. Most of the values were negative, indicating that the automated method generally had a constant error below the mean of the reference method. The highest proportional error observed in the automated method was 2.062, while the lowest was 0.001, representing the difference between the slope and its ideal value of 1.0. Out of the twenty-three organic acids, thirteen showed lower percentage bias in the automated method compared to the in-house method, indicating better accuracy with the automated approach.

However, there are some limitations and challenges associated with the described protocol. Firstly, the method is not fully online, as manual derivatization is still required. This is due to the corrosive nature of the derivatization reagents, which can damage the pipette heads of the Hamilton. Secondly, the number of samples that can be analyzed in a single run is limited. Thirdly, achieving complete separation between the aqueous and solvent phases was challenging since online centrifugation was not possible. Consequently, this resulted in longer mixing time, pipetting time, and waiting time to ensure clear separation between the two phases. It is important to avoid using a high concentration of hydrochloric acid, as it is corrosive, necessitating the cleaning of the pipette head after each run. Additionally, liquid classes should be optimized for each solvent to ensure accurate aspiration and dispensing on the Hamilton. Minimizing the time samples spend in the system is crucial to prevent loss due to evaporation, although the addition of an internal standard at the beginning of the run should compensate for any loss. Despite these challenges, the automated method was still relatively faster than the manual method, even though some steps had to be performed offline.

## 3. Materials and Methods

### 3.1. Chemicals and Materials

The following materials and chemicals were purchased from Merck, Johannesburg, South Africa: standards (98%) of glycolic acid, α-ketoglutaric acid, succinic acid, lactic acid, malonic acid, succinylacetone, glutaconic acid, adipic acid, methylmalonic acid, fumaric acid, ethylmalonic acid, phenyllactic acid, vanillylmandelic acid, 4-hydroxyphenylpyruvic acid, sebacic acid, 4-phenylbutyric acid, stearic acid, 3-methylglutaconic acid, phenylacetic acid, glutaric acid, citric acid, pyruvic acid, 3-phenylbutyric acid, and sorbitol, hexane, diethyl ether, and butanol, derivatization reagents, namely N-bis (trimethylsilyl) trifluoroacetamide (BSTFA) with trimethylchlorosilane (TMCS), and pyridine. Spectrometry-grade solvents, including methanol, acetonitrile, water, acetone, isopropyl alcohol, and ethyl acetate, were obtained from Honeywell Burdick & Jackson, Anatech, Johannesburg, South Africa. Sodium and magnesium sulphate were obtained from Merck, South Africa. Synthetic urine (SurineTM Lot 72110) was purchased from Dyna-tek industries, Johannesburg, South Africa. For validation experiments, ERNDIM External Quality Assurance Samples (EQAS) 2016 Quantitative Organic Acids samples were utilized. 

### 3.2. Preparation of Solutions

The organic acids were chosen based on their distinct physicochemical properties, such as volatility, pKa, solubility, and logP, to facilitate the development and validation of the method [15]. A working solution was prepared by combining each of the selected organic acids at a final concentration of 1 mg/mL for the standards. Additionally, sorbitol was included in the working solution to evaluate the extraction method’s selectivity. The internal standard, 3-phenylbutyric acid (3-PBA), was prepared separately at a final concentration of 0.52 mg/mL and stored at 4 °C. To create calibration standards, surine was spiked with the mixture of organic acid standards, resulting in concentrations ranging from 0 to 500 mg/L. 

### 3.3. Miniaturized Sample Preparation Procedure and Optimization

Prior to miniaturizing the extraction method, a series of optimization experiments were conducted. The method aimed to select two optimal solvents, one miscible and the other immiscible, for extracting organic acids from urine. After considering the literature and experimental data, acetonitrile was chosen as the initial one-phase extractant due to its polar nature and ability to extract polar compounds effectively. Ethyl acetate was selected as the immiscible solvent based on the experimental data and literature studies, showing good extraction efficiency and repeatability. The sample-to-solvent ratio was optimized, and the volumes of acetonitrile and ethyl acetate were reduced to 200 μL and 600 μL, respectively. To increase quantitative recoveries of organic acids, three cumulative sequential extractions with fresh organic solvent each time were performed. Pipette mixing was used to enhance surface contact between urine and solvents, and clear phase separations were achieved by allowing the samples to settle for 5 min. Drying the pooled top phase organic solvents with sodium sulfate facilitated quick evaporation and shortened the overall preparation time.

The miniaturized sample preparation method was adapted from the current in-house organic acid extraction method, referred to as the “in-house method” [16] (see Appendix A). To begin, 0.5 mL of urine was added to a 2 mL glass vial. Then, 100 μL of the internal standard (at a concentration of 0.52 mg/L) and 300 μL of acetonitrile were introduced into the vial. The samples were acidified with 4 drops of 5N HCl. Instead of using rotary mixing, pipette mixing was employed by repeatedly aspirating and dispensing the samples (10 times). After a 5 min interval, 600 μL of ethyl acetate was added, followed by another round of pipette mixing (10 times). The mixture was allowed to settle for 5 min to achieve clear phase separation. Subsequently, 600 μL of the organic solvent phase was aspirated into a second glass vial. The ethyl acetate extraction step was repeated twice. The collected sample was then dried at 40 °C using a gentle stream of nitrogen. The residue obtained was derivatized with 100 μL of BSTFA+TMCS (99:1) and 100 μL of pyridine and left to react at 60 °C for 45 min [17]. After approximately 5 min of cooling, the derivatized sample was transferred into inserts, and 1 μL of it was injected into the GC/MS. For evaluation of recovery rates, a consistent volume of the internal standard, BSTFA+TMCS, and pyridine was added, using the same concentration of the internal standard. 

### 3.4. Automation of Miniaturized Sample Preparation Protocol on a Hamilton Microlab STARlet (Hamilton Technologies)

After carefully selecting appropriate solvents, solvent volumes, and sample-to-solvent ratios through experimental trials, the miniaturization parameters were established as the basis for the automation method requirements. The miniaturized method was then translated into an automated extraction procedure using the ML Star Autosampler liquid handler. The Hamilton ML STAR is specifically designed for automated pipetting of liquid samples and reagents. Its deck is divided into equal tracks that guide carriers into predetermined positions. This particular Hamilton ML STAR model has one pipetting channel responsible for liquid transfer. The pipetting principle relies on air displacement, with each channel equipped with capacitive liquid level detection (cLLD) functionality. This enables the tip to detect the liquid surface during aspiration or dispensing by measuring the difference in capacitance between the tip in the air and the tip in the liquid.

To ensure high recovery of organic acids, the parameters for the workstations, including deck layout, speed, and positions of different aspiration and dispensing steps, were optimized. These optimizations were aimed at achieving a robust protocol capable of extracting up to 24 samples in a single run, even with volumes as low as 20 µL. It is important to note that the liquid Autosampler used for the method setup did not have the capability for online centrifugation.

**Figure 1:**
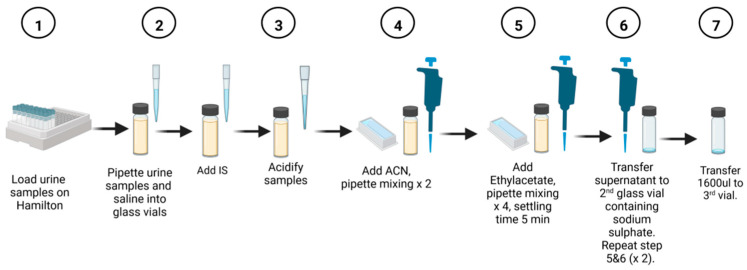
Scheme of the automated method. Steps 1–7 summarize the automated protocol on Hamilton ML STAR liquid handler. Besides manually loading the urine samples on the Hamilton, the rest of the steps were done automatically until step 7 when the vials were removed for offline drying and derivatization.

Before loading the samples onto the instrument, the creatinine value of each urine sample was determined. The programmed software then calculated the appropriate volume of urine to be pipetted based on the creatinine concentration in mmol/L. Figure 1 provides a schematic representation of the automated method. To normalize the samples for creatinine, they were diluted with normal saline to a maximum volume of 500 µL. The calculated amount of normal saline was then aspirated and dispensed into a 2 mL glass vial. Next, the determined urine volume was added to the saline. Based on the creatinine concentration, the internal standard (3-phenylbutyric acid) was pipetted into the sample vial. The sample was acidified with 50 µL of 5N HCl acid, followed by the addition of 200 µL of acetonitrile and two pipette mixing steps. The channel then aspirates 300 µL of the top phase and dispenses it at the bottom of the vial using high speed and pressure to ensure thorough mixing.

To maximize the extraction of organic acids, 600 µL of ethyl acetate was serially pipetted into the sample mixture, followed by four mixing steps. Each sample was allowed to stand for 5 min before transferring 600 µL of the top phase to the second vial. For the second extraction step with ethyl acetate, 700 µL was transferred to the second vial. To extract a greater quantity of organic acids, 300 µL of ethyl acetate was added to the sample without further mixing. This volume was then transferred to the second vial. The sample in the second vial was mixed again and left to stand for 5 min. Anhydrous sodium sulfate, added prior to the extraction protocol, effectively removed residual water from the sample. The final volume of the extracted sample was 1600 µL, which was transferred to a third rack using a clean glass vial. At this point, the automated extraction procedure was completed.

After completing the automated extraction procedure, the samples were removed from the liquid handler. They were then dried at 40 °C using a gentle stream of nitrogen. Next, the samples were derivatized by adding BSTFA/TMCS (99:1) and pyridine (4:1) in volumes determined based on the creatinine values. The derivatization reaction took place at 60 °C for 45 min. After the incubation period, the samples were allowed to cool for 5 min at room temperature. Finally, the derivatized samples were transferred to 2 mL glass vials in preparation for GC-MS analysis.

### 3.5. GCMS-Analysis

The GC-MS system utilized in this study was composed of an Agilent model 7890A gas chromatograph, a model 5975C mass selective detector, and an HP 5970C MS. A sample volume of one microliter was injected onto the GC column using split injection with a split ratio of 10:1 and a constant pressure of 8.2317 psi. The injection port temperature was set at 280 °C. The initial oven temperature was 50 °C and held for 1 min, followed by an increase to 60 °C at a rate of 2 °C/min, then to 120 °C at a rate of 5 °C/min, and finally to 295 °C at a rate of 7 °C/min, where it was held for 4 min. The total run time was 42.5 min, with an additional 2 min post-run at 300 °C. For separation, a fused-silica capillary column (DB-1MS UI, 30 m, 2.50 mm i.d., 0.25 µm film thickness) was utilized. Helium gas was used as the carrier gas at a constant flow rate of 1 mL/min. The MS source and quadrupole temperatures were set at 230 °C and 150 °C, respectively. Mass spectra of all GC peaks were generated by the mass spectrometer operating in the electron impact mode at 70 eV with SCAN (50–600 amu) positive ion monitoring.

### 3.6. Data Processing

The Automated Mass Spectral Deconvolution and Identification Software (AMDIS; Stein, 1999, The software is freely available on www.amdis.net) was employed to extract GC-MS data according to program specifications. The GC-MS data were analyzed in AMDIS using batch mode with the following deconvolution settings: component width of 20, adjacent peak subtraction of 1, resolution set to low, peak shape set to low, and sensitivity set to low. To minimize false positives, certain masses (TIC, 73, and 147) were excluded as model ions. An in-house organic acid library, developed based on mass spectral similarities (Reinecke et al., 2012), was utilized for compound identification. Compounds with low-confidence identification were removed, and peaks corresponding to the same compound were combined. Concentration values (mg/L) were determined using the internal standard, following the equation provided.

[A] = (RA/RIS) × [IS]

In the equation, [A] represents the concentration of analyte A, [IS] represents the concentration of the internal standard, RA represents the intensity value of analyte A, and RIS represents the intensity value of the internal standard. To assess the extraction efficiency, the concentration of each organic acid analyte was calculated for both pre-extraction and post-extraction spiked samples. The extracted organic acid analyte/IS ratio was then compared to the unextracted organic acid analyte/IS ratio. The recovery of each analyte was expressed as a percentage.

### 3.7. Method Equivalency between the Automated Extraction Method and the In-House Method

To assess the miniaturized extraction method compared to the in-house method, both absolute and relative recovery experiments were conducted using sorbitol and model organic acids. Afterward, the suitability of the automated method for use in a clinical laboratory was evaluated in terms of linearity, precision, and accuracy. Within-run and within-laboratory variations were calculated based on the obtained variance components.

To evaluate the performance of the automated method, synthetic “blank” urine samples were prepared at three different concentration levels: lower reference range, upper reference range, and a critical value. Each level was analyzed in triplicate per day for five consecutive days. Repeatability and within-laboratory precision were determined using the collected data, with the exclusion of outliers identified through the Cochran C and Dixon Q tests.

The accuracy of the automated organic acid extraction protocol was assessed by analyzing external quality assessment (EQA) samples obtained from ERNDIM. Triplicate samples from the same scheme year were analyzed and compared to the results obtained using the in-house method.

## 4. Conclusions

An optimized and fully automated urinary organic acid extraction method was implemented using the Hamilton ML Star AutoSampler. This automated method utilized a solvent-based extraction protocol for extracting organic acids from urine. It had a run time of approximately 30 min per sample and took around 3 h to process a batch of 24 samples. While it was based on the manually operated in-house extraction method, the automated method offered several advantages. These included rapid sample throughput, reduced labor intensity, minimized exposure to toxic solvents, elimination of exhaustive manual pipetting steps, short sample drying time, and the ability to process multiple samples within a single run. As a result, the analyst could allocate more time to analysis and data processing instead of sample preparation. The study successfully addressed the challenges associated with automating the solvent protocol, such as the addition of HCl, internal standard, centrifugation, and mixing. This enabled the complete automation of the extraction step in the overall sample preparation process.

## Figures and Tables

**Table 1:** Comparison of the in-house extraction protocol with the miniaturized one showing mean recovery% and CV%.

Organic Acids	Miniaturized Method %Mean Recovery	In-House Method %Mean Recovery	Miniaturized Method CV%	In-House Method CV%
Phenylacetic acid	135	122	4	9
Ethylmalonic acid	133	106	6	8
Methylmalonic acid	105	95	13	5
Stearic acid	102	95	3	8
4-Phenylbutyric acid	96	97	4	4
Fumaric acid	95	98	7	10
Adipic acid	76	89	9	8
Succinic acid	71	86	19	7
Glutaric acid	69	86	7	6
Lactic acid	46	105	10	24
Glutaconic acid	42	64	28	60
Glycolic acid	32	72	5	23
Citric acid	15	20	89	8
Sorbitol	2			

**Table 2:** Correlation coefficients and linear ranges for organic acids analyzed from 0 to 500 mg/L.

Organic Acids	Linear Range (mg/L)	Correlation Coefficient (r)
Phenylacetic acid	50–200	1
Fumaric acid	1–200	1
Sebacic acid	50–200	1
Stearic acid	1–300	1
Methylmalonic acid	1–300	0.999
Ethylmalonic acid	1–300	0.999
Adipic acid	1–300	0.999
Succinic acid	1–400	0.998
Glutaconic acid	1–300	0.998
Lactic acid	1–400	0.996
2-Ketoglutaric acid	1–300	0.996
Glutaric acid	1–400	0.995
Citric acid	1–300	0.995
4-Hydroxyphenylpyruvic acid	50–200	0.995
Succinylacetone	1–300	0.990
Malonic acid	1–500	0.988
Glycolic acid	1–500	0.981

**Table 3:** Method imprecision calculated as repeatability and within-laboratory precision.

	Repeatability			Within-Lab Precision		
Organic acids	50 mg/L	100 mg/L	500 mg/L	50 mg/L	100 mg/L	500 mg/L
Citric acid	15%	12%	7%	49%	41%	62%
Glycolic acid	23%	30%	8%	52%	42%	52%
Lactic acid	12%	8%	8%	29%	25%	32%
Succinic acid	3%	3%	6%	23%	14%	19%
Malonic acid	20%	7%	5%	42%	24%	54%
Fumaric acid	3%	7%	31%	18%	13%	29%
Glutaric acid	29%	18%	6%	29%	28%	21%
Glutaconic acid	14%	75%	85%	38%	86%	96%
Adipic acid	9%	14%	5%	26%	21%	19%
Methylmalonic acid	10%	8%	3%	27%	15%	32%
Ethylmalonic acid	3%	5%	28%	20%	13%	40%
4-Phenylbutyric acid	9%	4%	5%	26%	6%	6%
Stearic acid	11%	6%	12%	31%	19%	18%
Average	12%	15%	16%	32%	27%	37%

**Table 4:** Accuracy statistics from the method comparison plots for ERNDIM samples.

	Linear Regression (r)		Coefficient of the Slope (PE)		Y-Intercept (CE)	
Organic Acids	Automated Method	In-House Method	Automated Method	In-House Method	Automated Method	In-House Method
3-Methylglutaric acid	1	0.903	1.117	0.898	−0.732	5.392
Glutaric acid	1	0.956	0.933	0.806	−1.176	10.916
Sebacic acid	1	0.945	1.502	0.875	−0.528	3.831
Glyceric acid	0.999	0.950	0.09	0.077	−0.522	0.852
Ethylmalonic acid	0.999	0.798	1.052	1.49	−0.787	4.596
Glycolic acid	0.999	0.854	0.233	0.129	−2.849	−9.467
Suberic acid	0.999	0.870	1.352	0.913	−1.617	10.531
Methylmalonic acid	0.998	0.997	0.598	0.795	5.66	−3.018
N-Acetylaspartic acid	0.998	0.757	0.176	0.095	−0.275	−0.898
N-Tiglylglycine	0.998	0.838	0.5	0.55	−0.283	−1.217
3-Methylglutaconic acid	0.997	0.519	1.229	0.778	−1.272	19.777
3-Hydroxyisovaleric acid	0.997	0.349	0.779	0.033	−0.864	1.682
Fumaric acid	0.997	0.918	1.074	1.821	−1.011	−16.53
Adipic acid	0.995	0.913	1.037	1.847	−0.212	−22.759
3-Hydroxy-3-Methylglutaric acid	0.994	0.856	0.334	0.592	−5.716	9.049
Vanillactic acid	0.994	0.711	2.062	1.886	−0.732	−8.868
Methylcitric acid	0.991	0.426	0.324	0.059	−0.178	0.261
2-Hydroxyglutaric acid	0.990	0.635	0.506	0.286	−3.012	36.141
Pyroglutamic acid	0.989	0.766	0.173	0.273	−5.755	−16.759
Mevalonic acid	0.987	0.493	0.001	0.034	−0.021	2.03
N-Hexanoylglycine	0.985	0.735	0.542	0.624	0.231	−1.501
4-Hydroxybutyric acid	0.982	0.941	0.311	0.228	−1.982	−0.878
3-Hydroxyglutaric acid	0.614	0.614	−0.179	−0.152	0.966	0.82

## Data Availability

The data presented in this study are available on request from the corresponding author. The data are not publicly available due to being published in a thesis.

## Appendix A

The protocol was adapted from [16]. The urine volume used for organic acid analysis was determined according to the urinary creatinine values, based on the following guidelines:

- Creatinine values between 0.25 and 0.37 mmol/L: 3 mL of urine
- Creatinine values between 0.38 and 0.74 mmol/L: 2 mL of urine
- Creatinine values between 0.75 and 5.5 mmol/L: 1 mL of urine
- Creatinine values of and above 5.56 mmol/L: 0.5 mL of urine

Urine samples were transferred to silanized glass tubes (Kimax), and internal standard (3-phenylbutyric acid) was added to the tubes to a final concentration of 180 mmol/mol creatinine. The samples were then acidified with 6 drops of 5N HCl. Six ml of ethyl acetate was added to the sample mixture and mixed on a rotary wheel for 30 min. The mixture was centrifuged at 3000 revolutions per minute (rpm) for 3 min; next, the organic phase was carefully aspirated into a second tube. Three ml of diethyl ether was then added to the remaining aqueous phase in the first kimax tube and mixed on the rotary wheel for 10 min. After centrifugation (3000 rpm, 3 min), the organic phase was aspirated into the second kimax tube containing the ethyl acetate extract. A small amount of sodium sulphate was added to the second kimax tube. This mixture was vortexed and centrifuged (3000 rpm, 3 min) and transferred to a clean tube. The extracted organic phase was evaporated to dryness under nitrogen at 40 °C. The organic acid residue was derivatized by adding BSTFA/TMCS (99:1) and pyridine (5:1, volume added is based on the creatinine values). The samples were incubated for 45 min at 60 °C, allowed to cool for 5 min at room temperature, and transferred to a 2 mL glass vial for GC-MS analysis.

## References

[1] Blau N., Duran M., Michael Gibson K., Vici C.D. (2014). Physician’s Guide to the Diagnosis, Treatment, and Follow-Up of Inherited Metabolic Diseases.

[2] Kauna-Czaplińska J. (2011). Current Applications of Gas Chromatography/Mass Spectrometry in the Study of Organic Acids in Urine. Crit. Rev. Anal. Chem..

[3] Jones P.M., Bennett M.J. (2010). Urine Organic Acid Analysis for Inherited Metabolic Disease Using Gas Chromatography-Mass Spectrometry. Methods Mol. Biol..

[4] Goodman S.I., Markey S.P. (1981). Diagnosis of Organic Acidemias by Gas Chromatography-Mass Spectrometry. Lab. Res. Methods Biol. Med..

[5] Hoffmann G.F., Feyh P., Blau N., Duran M., Blaskovics M.E., Gibson K.M. (2003). Organic Acid Analysis. Physician’s Guide to the Laboratory Diagnosis of Metabolic Diseases.

[6] Raterink R.-J., Lindenburg P.W., Vreeken R.J., Ramautar R., Hankemeier T. (2014). Recent Developments in Sample-Pretreatment Techniques for Mass Spectrometry-Based Metabolomics. Trends Analyt. Chem..

[7] Tanaka K., West-Dull A., Hine D.G., Lynn T.B., Lowe T. (1980). Gas-Chromatographic Method of Analysis for Urinary Organic Acids. II. Description of the Procedure, and Its Application to Diagnosis of Patients with Organic Acidurias. Clin. Chem..

[8] Álvarez-Sánchez B., Priego-Capote F., Castro M.D.L. (2010). de Metabolomics Analysis II. Preparation of Biological Samples prior to Detection. Trends Analyt. Chem..

[9] Lord H.L., Pfannkoch E.A. (2012). Sample Preparation Automation for GC Injection. Comprehensive Sampling and Sample Preparation.

[10] Clement R.E., Hao C. (2012). Liquid–Liquid Extraction. Comprehensive Sampling and Sample Preparation.

[11] Ford B.A., McElvania E. (2020). Machine Learning Takes Laboratory Automation to the Next Level. J. Clin. Microbiol..

[12] Wilson S., Steele S., Adeli K. (2022). Innovative Technological Advancements in Laboratory Medicine: Predicting the Lab of the Future. Biotechnol. Biotechnol. Equip..

[13] Kebede T.G., Nety S.S., Dube S., Nindi M.M. (2022). The miniaturization of liquid-phase extraction techniques. Emerging Freshwater Pollutants: Analysis, Fate and Regulations.

[14] Kannouma R.E., Hammad M.A., Kamal A.H., Mansour F.R. (2022). Miniaturization of Liquid-Liquid extraction; the barriers and the enablers. Microchem. J..

[15] Phiri M.M. (2016). Re-assessment and optimisation of an organic acid extraction method for automation. Biochemistry.

[16] Reinecke C.J., Koekemoer G., van der Westhuizen F.H., Louw R., Lindeque J.Z., Mienie L.J., Smuts I. (2012). Metabolomics of Urinary Organic Acids in Respiratory Chain Deficiencies in Children. Metabolomics.

[17] Christou C., Gika H.G., Raikos N., Theodoridis G. (2014). GC-MS Analysis of Organic Acids in Human Urine in Clinical Settings: A Study of Derivatization and Other Analytical Parameters. J. Chromatogr. B Analyt. Technol. Biomed. Life Sci..

